# Microwave Radiation Caused Dynamic Metabolic Fluctuations in the Mammalian Hippocampus

**DOI:** 10.3390/metabo14070354

**Published:** 2024-06-23

**Authors:** Yu Xin, Shu-Ting Guan, Ke Ren, Hui Wang, Ji Dong, Hao-Yu Wang, Jing Zhang, Xin-Ping Xu, Bin-Wei Yao, Li Zhao, Chang-Xiu Shi, Rui-Yun Peng

**Affiliations:** 1 School of Education, Hebei University, Baoding 071002, China; xinyumeow@gmail.com (Y.X.); okayke1@163.com (K.R.); 2 Institute of Radiation Medicine, Beijing 100850, China; guanst59@163.com (S.-T.G.); wanghui597bj@163.com (H.W.); djtjwj@163.com (J.D.); smart106@126.com (H.-Y.W.); zhang115614@163.com (J.Z.); xxpbjhd@163.com (X.-P.X.); ybwcsq@163.com (B.-W.Y.)

**Keywords:** microwave radiation, hippocampus injury, metabolomics

## Abstract

To investigate the dynamic changes in hippocampal metabolism after microwave radiation using liquid chromatography in tandem with mass spectrometry/mass spectrometry (LC–MS/MS) and to identify potential biomarkers. Wistar rats were randomly assigned to a sham group and a microwave radiation group. The rats in the microwave radiation group were exposed to 2.856 GHz for 15 min for three times, with 5 min intervals. The rats in the sham group were not exposed. Transmission electron microscope revealed blurring of the synaptic cleft and postsynaptic dense thickening in hippocampal neurons after microwave radiation. Metabolomic analysis revealed 38, 24, and 39 differentially abundant metabolites at 3, 7, and 14 days after radiation, respectively, and the abundance of 9 metabolites, such as argininosuccinic acid, was continuously decreased. After microwave radiation, the abundance of metabolites such as argininosuccinic acid was successively decreased, indicating that these metabolites could be potential biomarkers for hippocampal tissue injury.

## 1. Introduction

Microwave technology is widely used in modern technology, such as medical equipment, communication systems, household appliances, military instruments, geological exploration, meteorological measurement systems, and disinfection and sterilization equipment. Although microwave technology has increased productivity and quality of life, it has also become an important source of pollution, and it is beginning to receive increasing attention. Studies have shown that microwave radiation can cause damage to multiple systems, such as the nervous system, reproductive system, immune system, and respiratory system. The nervous system is a target of microwave radiation exposure since it is the excitable component of the body that interacts with electromagnetic fields [1]. The earlier reports showed that 2.856 GHz microwave radiation could induce hippocampal structural damage, and spatial memory and recognition memory declined. After radiation, the nucleus of hippocampal neurons was pyknotic and hyperchromatic, and the content of Nissl bodies was reduced [2]. In addition, the secretion of amino acid transmitters in the hippocampus of rats after 2.856 GHz microwave radiation is imbalanced, which may be related to the change in the metabolic profile [3]. However, the potential regulatory mechanisms in metabolomics have not been clarified.

The hippocampus is sensitive to microwave radiation [3,4,5] and has dense neurons and synaptic connections. In addition, the hippocampus is closely involved in the activities of the nervous system. It not only has an internal trisynaptic circuit (CA1-CA3-dentate gyrus) [6] but also interacts with multiple upstream and downstream brain regions, such as the inner and outer olfactory cortex, amygdala, and the locus coeruleus [7,8,9]. The hippocampus participates in a series of advanced cognitive functions, such as information coding computation, memory retrieval, prospective cognition, and emotional regulation [10,11,12]. Studies have shown that hippocampal tissue damage is closely related to memory decline, physical disorders such as depression and anxiety, and neurodegenerative diseases such as Alzheimer’s disease [12,13].

The brain, as the central organ governing body functions, is abundant in biological metabolites. The synthesis and metabolism of these compounds play crucial roles in the functioning of the central nervous system. Metabolites such as lipids act on the signal transmission and regeneration process of neurons through metabolic pathways [14]. Disturbances of substances such as sugars and lipids have been proven to affect energy supplies and cause cognitive impairment [15]. Targeting brain metabolism may be a therapeutic strategy for the treatment of functional injury and degeneration. Microwave radiation can cause energy metabolism disorders and metabolic spectrum changes in the body [16,17], but the relevant signaling pathways and biomarkers are not yet known.

In this study, liquid chromatography–mass spectrometry/mass spectrometry (LC–MS/MS) was used to explore the dynamic changes in the metabolic spectra of the rat hippocampus after microwave exposure. The metabolites sensitive to the hippocampal synaptic damage induced by microwave radiation in rats were identified, and their metabolic pathways were identified, providing a basis for the prevention and treatment of microwave radiation-induced cognitive damage.

## 2. Materials and Methods

### 2.1. Animal Grouping and Microwave Radiation Methods

Male Wistar rats (180–220 g) from Beijing Vitong Lever Company (Beijing, China) were randomly divided into two groups: the microwave radiation (MW) group and the sham radiation (sham) group. They were tested on days 3, 7, and 14 after radiation. The animals were allowed access to food and water and were housed under a 12 h/12 h light/dark cycle. The present procedure for the disposing of animals conforms to the requirements set forth by the Ethics Committee of the Institute of Radiation Medicine (review number: IACUC-DWZX-2020-610). The rats were exposed to total body radiation using microwave radiation equipment at the institutions. Cone-shaped absorbers were placed throughout the protected space to lessen reflections. The microwave radiation frequency was 2.856 GHz, the average power density was 30 mw/cm^2^, and the radiation time was three times in 15 min, with 5 min intervals. The sham group received the same treatment as the MW group, except that they were not exposed to microwave radiation.

### 2.2. Transmission Electron Microscopy (TEM) Observation of Hippocampus

A total of 18 6-week-old rats were used for TEM sample observation, with three rats from each group. All animals were anesthetized with 1% pentobarbital sodium. Hippocampal tissue was isolated from the rat brain at 3, 7, and 14 days after microwave radiation. Next, a 1 mm^3^ tissue block was obtained with a blade, stored at 4 °C with 3% glutaraldehyde, and then put into PBS supplemented with 0.19 mol/L sucrose buffer. The tissue was fixed with 1% osmic acid and then washed with PBS plus 0.19 mol/L sucrose buffer. After dehydration using alcohol gradients, the tissue was submersed in acetone and an embedding agent, and 1.5 μm semithin sections of tissue samples were prepared for hematoxylin–eosin (HE) staining. Then, the sections were stained with uranyl acetate staining solution and lead nitrate staining solution and observed by transmission electron microscopy (HT7800, Hitachi, Tokyo, Japan).

### 2.3. LC–MS/MS

#### 2.3.1. Sample Collection and Processing

A total of 27 samples were used for LC-MS/MS. Hippocampal tissue from the rat brain was isolated at 3, 7, and 14 days after microwave radiation. Around 100 mg of the sample was frozen with liquid nitrogen and ground. Three times the volume of the lysis buffer (80% methanol) was added, the mixture was vortexed, and the tissue disruptor was ground at 45 Hz for 4 min, followed by ultrasonication (in an ice water bath) for 5 min. After 30 min of standing at −20 °C and 30 min of centrifuging at 14,000× *g*, the supernatant was collected and filtered through a 0.45 μm filter, and 20 μL of the supernatant was placed in a sampling bottle for detection. For machine testing, a similar volume of supernatant was extracted from each sample and combined with quality control (QC) samples.

#### 2.3.2. Chromatographic and Mass Spectrometric Parameters

The chromatographic parameters were as follows: mobile phase A was acetonitrile/water (60/40), mobile phase B was isopropyl alcohol/acetonitrile (90/10), and both A and B contained 0.1% formic acid and 10 mmol/L ammonium formate. The sample volume was 5 μL, and the sample was loaded using the sandwich method. The loading pump flow rate was 0.25 mL/min for 20 min. The gradient elution program was performed as follows: 0 min 98% B;1 min 98% B; 5 min 30% B; 8 min 0% B; 14 min 0% B; and 16 min 98% B.

The mass spectrometry parameters were as follows: the ion source voltage was set to 3.5 kV, and the heating temperature was 300 °C. The full mass scan parameters were as follows: resolution 70,000, automatic gain control target below 3 × 10^6^, maximum isolation time 100 ms, and 100–1500 *m*/*z*. The MS/MS scan parameters were as follows: resolution 17,500, automatic gain control target below 1 × 10^5^, maximum isolation time 50 ms, and normalized collision energies of 10, 30, and 60 V.

#### 2.3.3. Data Analysis

The postsynaptic density of the rats was quantitatively analyzed using ImageJ 2.1.0 (National Institutes of Health, Bethesda, MD, USA). SPSS 25.0 (International Business Machines Corporation, Armonk, NY, USA) was used to analyze the experimental data. The independent samples *t*-test was used for statistical analysis. A significant difference between the two groups was indicated by a *p* value lower than 0.05. Figures were drawn with GraphPad Prism 9.5.1 (GraphPad Software, La Jolla, CA, USA) and Adobe Illustrator 28.0 (Adobe, Atlanta, GA, USA).

The MS data were analyzed using Progenesis QI 3.0 (Nonlinear Dynamics, Newcastle upon Tyne, UK). Data alignment was performed for different samples according to a retention time deviation of 0.2 min and a mass deviation of 5 ppm. The coefficient of variation (CV) was 30%, the signal-to-noise ratio was 3, and the minimum signal intensity was 100,000. We obtained the crude data sets comprising peak names, sample information, peak area, retention time, and quality score ratio. After deleting all the pseudo-positive peaks, the data were normalized. The peaks of the same compounds were combined. Finally, the identification and quantification results of metabolites were obtained. The differentially expressed metabolites in the MW group compared to the sham group samples were identified according to the independent sample *t*-test. The differentially expressed metabolites between groups were screened and considered significant (fold change ≥ 2 or < 1/2, *p* value < 0.05, VIP ≥ 1). In addition, all differentially abundant metabolites were subjected to mapping in the KEGG database (Kyoto Encyclopedia of Genes and Genomes) to gather relevant information and gain insights into the changes in metabolism pathways. The discriminative ability of candidate biomarkers was assessed by the AUC produced by receiver operating characteristic (ROC) curves.

## 3. Results

### 3.1. Ultrastructural Damage to Synapses in the Rat Hippocampus after Microwave Radiation

**Figure 1:**
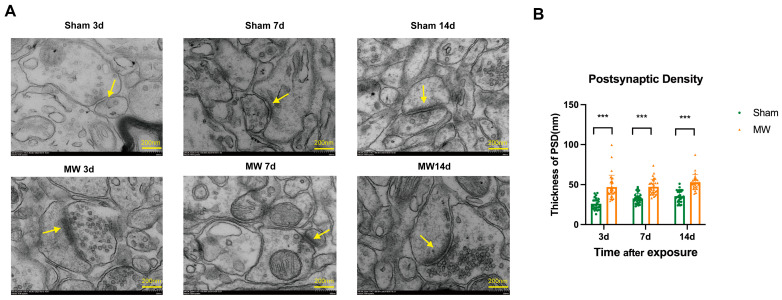
Ultrastructural changes in rat synapses after microwave irradiation (TEM, *N* = 3 in each group, scale bar = 200 nm). (**A**) Synaptic structure of rat hippocampal tissue; (**B**) quantitative analysis of postsynaptic density in rat hippocampal tissue. The yellow arrow points to the postsynaptic density. *** shows *p* < 0.001.

We used TEM to observe synaptic ultrastructures that are closely related to memory function. TEM revealed that at 3, 7, and 14 days after radiation, compared with those in the sham group, the rats in the MW group exhibited structural damage, such as the accumulation of presynaptic vesicles, blurring of the synaptic cleft, and thickening of the postsynaptic density in the hippocampal neurons (Figure 1A). In order to measure the postsynaptic density, a unified magnification of 40× was used for image shooting. We took more than 20 photos for quantitative statistics. At least 24 postsynaptic densities were measured in each group. The quantitative results revealed that the postsynaptic density was significantly greater (Figure 1B, *p* < 0.001). The above results suggest that microwave radiation can cause damage to synaptic structures in hippocampal tissue.

### 3.2. Perturbations in the Metabolic Profile of Rat Hippocampal Tissue after Microwave Radiation

Early studies based on the same radiation parameter reported the memory impairment induced by microwave radiation [2]. Based on the damage to spatial memory and synaptic structures in rats after microwave radiation, we used LC/MS-MS to observe whether the metabolic profile of the rat hippocampus changed after microwave radiation to explore potential biomarkers.

#### 3.2.1. QC Sample Consistency Assessment and Model Fitting Identification

**Figure 2:**
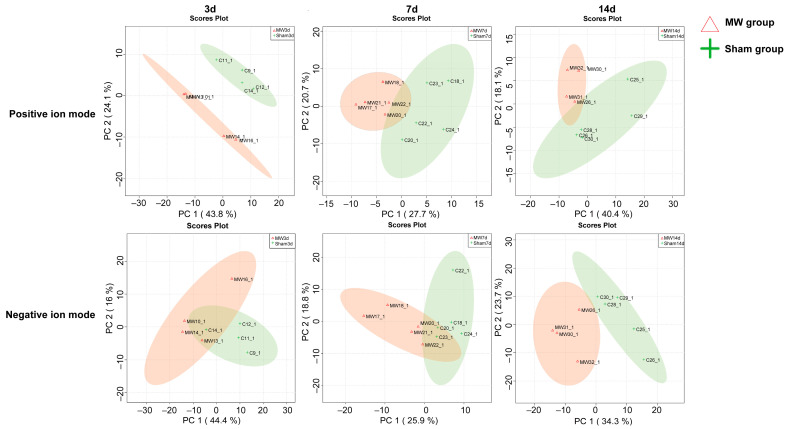
A PCA diagram in positive ion mode and negative ion mode at 3 d, 7 d, and 14 d after microwave radiation. Horizontal coordinates PC1 and vertical coordinates PC2 represent the scores of the first and second principal components, respectively. Scattered dots of different colors represent samples from different experimental groups, and the ellipse is a 95% confidence region. The red triangle is the sample of the microwave radiation group, and the green + is the sample of the sham group.

According to the results of the QC sample test, there was suitable consistency between the samples, with a consistency coefficient greater than 0.95. The PCA results showed that samples within the same group were clustered and that there was a clear separation between the groups. This suggests that the samples within the same group had suitable reproducibility and that there was excellent group discrimination. The metabolic profile of rat hippocampal tissue affected by microwave radiation fluctuated (Figure 2).

#### 3.2.2. Differentially Abundant Metabolite Screening and Annotation

##### Notes on Differentially Abundant Metabolite Clusters

**Figure 3:**
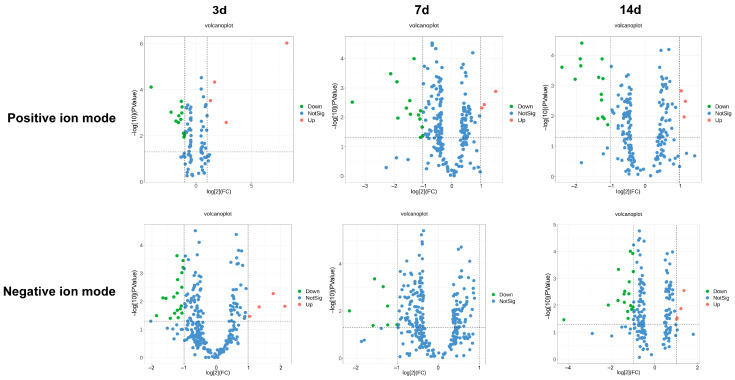
A volcano plot of metabolite changes in positive ion mode and negative ion mode after 3 d, 7 d, and 14 d of microwave radiation. The horizontal dotted line indicates that *p* = 0.05 is the dotted line corresponding to the logarithm, and the vertical dotted line indicates that the FC threshold is the dotted line corresponding to the logarithm. Points that are above the horizontal dashed line and on both sides of the vertical dashed line are highlighted. The red highlight on the right means up, and the green highlight on the left means down. The blue dot indicates that it has not reached the standards.

Based on the criteria (FC ≥ 2 or < 1/2, *p* ≤ 0.05, VIP ≥ 1), positive and negative ion modes were used to assess a total of 101 differentially abundant metabolites. The differentially abundant metabolites mainly included lipid and lipid molecules, organic heterocyclic compounds, organic oxygen compounds, phenylpropanoids and polyketones, organic acids and their derivatives, and alkaloids. On the 3rd day after microwave radiation, the abundance of 8 metabolites was significantly increased (*p* < 0.05), and the abundance of 30 metabolites was significantly decreased (*p* < 0.05). On the 7th day after microwave radiation, the abundance of 3 metabolites was significantly increased (*p* < 0.01), and the abundance of 21 metabolites was significantly decreased (*p* < 0.05). On the 14th day after microwave radiation, the abundance of 7 metabolites was significantly increased (*p* < 0.05), and the abundance of 32 metabolites was significantly decreased (*p* < 0.05) (Figure 3).

The abundance of nine different metabolites was continuously decreased over the 3rd, 7th, and 14th days after microwave radiation, (*p* < 0.05). The receiver operating characteristic (ROC) curve was used to evaluate the performance of the above metabolites in predicting events. The area under the curve (AUC) values were all ≥0.90, indicating that these molecules have suitable predictive power. Moreover, the abundance of bergamol and 2-chloro-5-methylmaleylacetate was continuously decreased on the 7th and 14th days after microwave irradiation, and these metabolites also exhibited suitable predictive ability (AUC ≥ 0.95). These metabolites may be sensitive to hippocampal tissue injury caused by microwave radiation (Table 1).

#### 3.2.3. Analysis of Differential Metabolic Pathways

KEGG pathway enrichment analysis of the differentially abundant metabolites captured at each time point revealed that 16 metabolic pathways were enriched (Figure S1 in Supplementary Materials). There are several pathways associated with cognitive processes. Quinic acid is involved in the phenylalanine, tyrosine, and tryptophan biosynthesis pathways. Argininosuccinic acid is involved in arginine synthesis and alanine, glutamic acid, and aspartic acid metabolic pathways. Lysophosphatidylcholine (14:1(9Z)/0:0) is involved in phospholipid metabolism and the choline metabolism pathway in tumor cells. 5-Methylthioribose is involved in cysteine and methionine metabolism and other pathways. Bergamol is involved in the PPA biosynthesis pathway.

## 4. Discussion

Microwave radiation has been found to affect neurotransmitters; cause neuronal DNA damage, neuronal degeneration, and apoptosis; and lead to neurodegenerative diseases. It has also been shown to damage the hippocampus and synaptic plasticity and even cause behavioral changes such as anxiety and memory dysfunction [18,19,20]. The hippocampus has high metabolic activity, and microwave radiation induces metabolic disorders of the nervous system. Disruptions to metabolic pathways can impact both normal and pathological processes. Metabolomics focuses on the dynamic changes in the quantity and quality of endogenous metabolites caused by external stimuli and can detect changes in the metabolic profile and interactive mapping relationships from a holistic perspective [21,22].

This study examined the dynamic changes in the metabolic profile of hippocampal tissues after microwave radiation exposure via LC–MS/MS technology. Argininosuccinic acid, lysophosphatidylcholine, bergamol, and other metabolites exhibited significant changes and high predictive value in the model. These metabolites may be potential indicators of hippocampal tissue injury caused by microwave radiation. Changes in metabolic pathways may be involved in the physiological mechanism of hippocampal injury and cognitive degeneration after microwave radiation.

The abundance of arginine succinic acid, lysophosphatidylcholine, and arachidyl trifluoromethyl ketone was continuously and significantly decreased after microwave radiation, and related studies revealed that these metabolites are involved in cognitive processes. Argininosuccinic acid is a metabolite of the urea cycle. Its precursors are aspartic acid and citrulline, which are subsequently decomposed into arginine and fumaric acid. The loss of activity of related enzymes leads to an abnormal content of argininosuccinic acid in body fluids, cells, or tissues [23]. Argininosuccinic acid metabolism is closely related to cognition, and the loss of argininosuccinic acid synthase leads to the harmful accumulation of ammonia in the blood and cerebrospinal fluid and impairs brain function [24,25]. Some studies have shown that excess lysophospholipids can lead to demyelination and memory impairment [26]. However, after intervention with medium-chain triglycerides (MCTs) in AD patients, the lysophosphatidylcholine levels increased, and cognitive ability improved [27]. Arachidonic trifluoromethyl ketone can inhibit the elevated activity of GIVA-PLA2 in familial AD, and its intervention can improve Aβ-induced neurotoxicity, providing a reference for prevention and treatment [28]. In addition, we found that the abundance of bergamol was also continuously decreased 14 days after microwave radiation. Some studies have confirmed that bergamol has anticancer, anti-inflammatory, and antiatherosclerotic medicinal properties. In a neuroinflammation model induced by lipopolysaccharide exposure, bergamol treatment significantly improved cognitive impairment, hippocampal dendritic spine injury, and the expression of inflammatory factors [29]. In summary, a number of studies have shown that metabolites such as arginine succinic acid, lysophosphatidylcholine, and arachidyl trifluoromethyl ketone participate in the operation and repair of cognitive function. The continuous decrease in the abundance of these metabolites after microwave radiation may be an important cause of hippocampal structural and functional injury and cognitive degeneration. These metabolites may be potential sensitive indicators of hippocampal injury after microwave radiation.

Amino acid metabolism is involved in the synthesis of neurotransmitters and neuromodulators that are directly or indirectly involved in synaptic transmission and learning and memory processes in the brain [10]. Lipids are essential for the normal development and function of the central nervous system, and impaired lipid metabolism is regarded as a key factor in the pathogenesis of neurodegenerative diseases [30]. Multiple cognitively relevant amino acid synthesis and metabolic pathways are altered after microwave radiation. The aromatic amino acids tryptophan, tyrosine, and phenylalanine are biosynthetic precursors of the neurotransmitters serotonin, dopamine, and norepinephrine [31]. Aspartate and glutamate are highly expressed in the brain and are beneficial for learning and memory [32]. Cysteine can improve memory impairment and oxidative stress and restore neurotransmission in animals [33,34]. Dysregulation of lysophosphatidylcholintransferase 1, a key enzyme in the phospholipid metabolic pathway, is directly related to learning and memory deficits [35]. In this study, the differential changes in these pathways may be a pathological mechanism of microwave-induced spatial memory dysfunction in rats.

## 5. Conclusions

In summary, this study revealed that ultrastructural damage to synapses and metabolic profile changes were induced in the rat hippocampus by microwave radiation, and it can take part in spatial memory dysfunction. A total of 101 differentially abundant metabolites were found to be mainly involved in amino acid metabolism and phosphatidyl metabolism, and 9 metabolites, such as arginine succinic acid, may be potential sensitive biomarkers.

## Figures and Tables

**Table 1:** Analysis of potentially sensitive metabolites to microwave radiation.

Ionization Mode	Metabolite	HMDB ID	Super-Class	Chemical Formula	Trend	VIP	*p* Value	AUC
+	Lysophosphatidylcholine (14:1(9Z)/0:0)	HMDB0010380	Lipids and lipid-like molecules	C_22_H_44_NO_7_P	Down	>5	<0.01	1
+	Argininosuccinic acid	HMDB0000052	Organic acids and derivatives	C_10_H_18_N_4_O_6_	Down	>2	<0.05	≥0.92
+	Aacocf3	HMDB0247736	\	C_21_H_31_F_3_O	Down	>4	<0.01	1
−	fleroxacin	HMDB0252299	Organoheterocyclic compounds	C_17_H_18_F_3_N_3_O_3_	Down	>3	<0.05	≥0.92
+	3-Cyclohexyl-1-propylsulfonic acid	HMDB0244400	Organic nitrogen compounds	C_9_H_19_NO_3_S	Down	>2	<0.01	1
+	17a-Ethynylestradiol	HMDB0001926	Lipids and lipid-like molecules	C_20_H_24_O_2_	Down	>2	<0.05	≥0.95
+	Inuline	HMDB0248466	Lipids and lipid-like molecules	C_32_H_46_N_2_O_8_	Down	>2	<0.01	1
−	Bakkenolide D	HMDB0034998	Lipids and lipid-like molecules	C_21_H_28_O_6_S	Down	>2	<0.01	1
−	2-Aminoanthracene	HMDB0245007	Benzenoids	C_14_H_11_N	Down	>2	<0.05	1
+	Bergaptol	HMDB0013679	Phenylpropanoids and polyketides	C_11_H_6_O_4_	Down	>2	<0.05	≥0.95
+	2-Chloro-5-methylmaleylacetate	HMDB0060346	Organic acids and derivatives	C_7_H_7_ClO_5_	Down	>2	<0.05	≥0.95

VIP (Variable Importance in the Projection) shows the contribution rate of differential metabolites. AUC (area under the curve) shows the effect of each potential metabolite induced independently. The + means the metabolite changes in positive ion mode, and the − means in negative ion mode.

## Data Availability

The data can be accessed through the corresponding authors when the reasons are appreciated.

## Supplementary Materials

The following supporting information can be downloaded at: https://www.mdpi.com/article/10.3390/metabo14070354/s1, Figure S1: KEGG pathways of the differentially abundant metabolites captured in positive and negative ion modes at 3 d, 7 d and 14 d after microwave radiation.
