# Supplementary material for: Microwave Radiation Caused Dynamic Metabolic Fluctuations in the Mammalian Hippocampus
Source: Metabolites. 2024 Jun 23;14(7):354. doi: 10.3390/metabo14070354 (PMC11278544; doi:10.3390/metabo14070354)
Supplement: Supplementary file 1 [file metabolites-14-00354-s001.zip › metabolites-2993342-supplementary.pdf]

metabolites

Microwave Radiation Caused Dynamic Metabolic Fluctuations in the Mammalian Hippocampus

Yu Xin [1,2], Shu-Ting Guan [2], Ke Ren [1,2], Hui Wang [2], Ji Dong [2], Hao-Yu Wang [2], Jing Zhang [2], Xin-Ping Xu [2], Bin-Wei Yao [2], Li Zhao [2,*], Chang-Xiu Shi [1,*] and Rui-Yun Peng [1,2,*]

1 School of Education, Hebei University, Baoding 071002, China; xinyumeow@gmail.com (Y.X.); okayke1@163.com (K.R.)
2 Institute of Radiation Medicine, Beijing 100850, China; guanst59@163.com (S.-T.G.); wanghui597bj@163.com (H.W.); djtjwj@163.com (J.D.); smart106@126.com (H.-Y.W.); zhang115614@163.com (J.Z.); xxpbjhd@163.com (X.-P.X.); ybwcsq@163.com (B.-W.Y.)
* Correspondence: lillyliz@163.com (L.Z.); scxsci@outlook.com (C.-X.S.); pengry@bmi.ac.cn (R.-Y.P.)

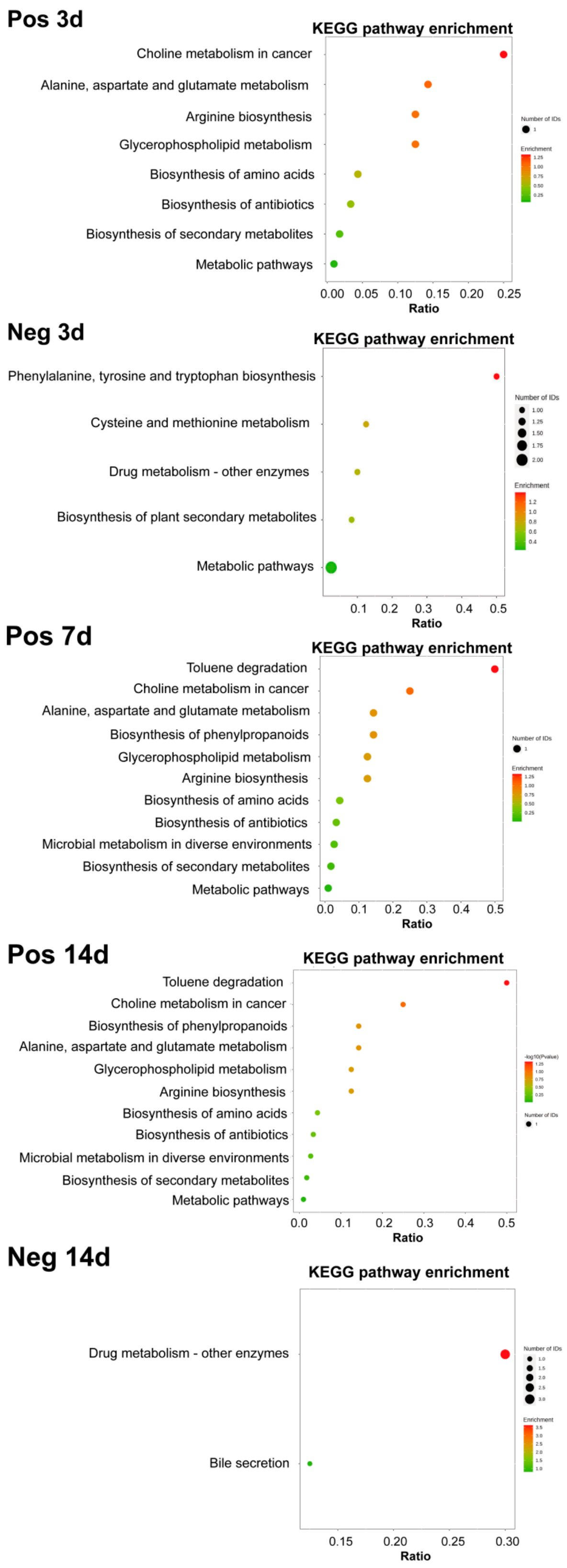

Figure S1. KEGG pathways of the differentially abundant metabolites captured in positive and negative ion modes at 3 d, 7 d and 14 d after microwave radiation.
